# A Case of a Ventricular Septal Defect (VSD) Leading to a Left Ventricular Aneurysm

**DOI:** 10.7759/cureus.25781

**Published:** 2022-06-09

**Authors:** Matheus Moreira, George B Walbridge, Narden Gorgy, Dennis Ehrich

**Affiliations:** 1 Internal Medicine, Touro College of Osteopathic Medicine, Syracuse, USA; 2 College of Osteopathic Medicine, University of New England, Syracuse, USA; 3 Cardiology, St. Joseph's Hospital, Syracuse, USA

**Keywords:** ventricular septal defect (vsd), bidirectional shunt, basal inferior aneurysm, myocardial infarction, congestive heart failure

## Abstract

Ventricular septal defect (VSD) and left ventricular aneurysm can be indicative of a previous myocardial infarction (MI). Although VSDs and aneurysms are common from MIs the possibility remains of their formation from mechanical complications in the heart. This case reports an unusual finding of a VSD and a left ventricle aneurysm without subjective findings of a previous MI or a past history of any ischemic event.

## Introduction

Acquired ventricular septal defects (VSDs) are rare in nature and normally occur in less than 1% of cases of myocardial infarction (MI) [1]. Post-MI VSDs are most commonly caused by an occlusion of the left anterior descending artery. However, occlusion of any vessels that provide septal branches, without the presence of collaterals, has the potential to form a VSD [1-2]. Depending on the severity, the lack of perfusion to the interventricular septum during an MI results in tissue destruction and the possible formation of a septal defect [3].

Up to 85% of left ventricular aneurysms are located in the anterior wall due to left anterior descending coronary artery occlusion. Only 15% of left ventricular aneurysms are associated with a right coronary artery (RCA) infarct affecting the posterior inferior wall, as seen in the patient presented in this case [4-5].

## Case presentation

A 66-year-old male with a past medical history of schizoaffective disorder, type II diabetes mellitus, chronic obstructive pulmonary disease, hypertension, and no known genetic disorders, with a 68-pack-year smoking history presented to the emergency department from home with bilateral leg swelling for the past three months, and new-onset shortness of breath at rest. The patient was unable to perform his daily activities of living or able to lie flat. His hygiene worsened over the past three months prompting his son to activate emergency services. The patient denied any chest pain, pressure, nausea, fever, chills, or sweats.

Physical examination on admission was significant for hypotension of 93/66, a heart rate of 96 bpm, increased jugular venous pressure, a grade 2 systolic ejection murmur of the second right intercostal space, bilateral rales and rhonchi on auscultation, and anasarca with 4+ pitting edema of his legs, scrotal edema, and abdominal edema.

**Figure 1:**
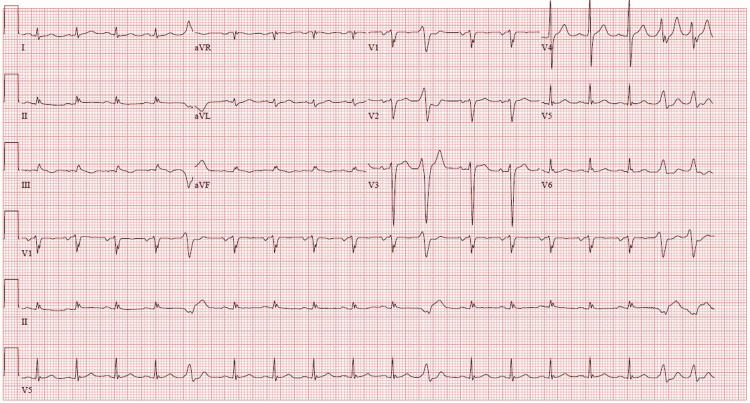
Initial EKG on admission. EKG showing sinus tachycardia with frequent premature ventricular contractions, low voltage QRS, and nonspecific T wave abnormalities.

Point of care Troponin I and subsequent Troponin I was found to be slightly elevated at 0.05 ng/mL and 0.06 ng/mL respectively, with normal being 0.00-0.04 ng/mL. His N-terminal proBNP was elevated at 13,694 pg/mL. On admission, the patient was hyponatremic with a sodium of 130 mEq/L (normal 135-145 mEq/L) and hyperkalemic with a potassium of 5.4 mmol/mL (normal 3.6-5.2 mmol/mL). All other electrolytes were unremarkable. Initial 12 lead EKG seen in Figure 1 showed sinus tachycardia with frequent premature ventricular contractions, low voltage QRS, and nonspecific T wave abnormalities. The patient was diagnosed with new-onset congestive heart failure.

**Figure 2:**
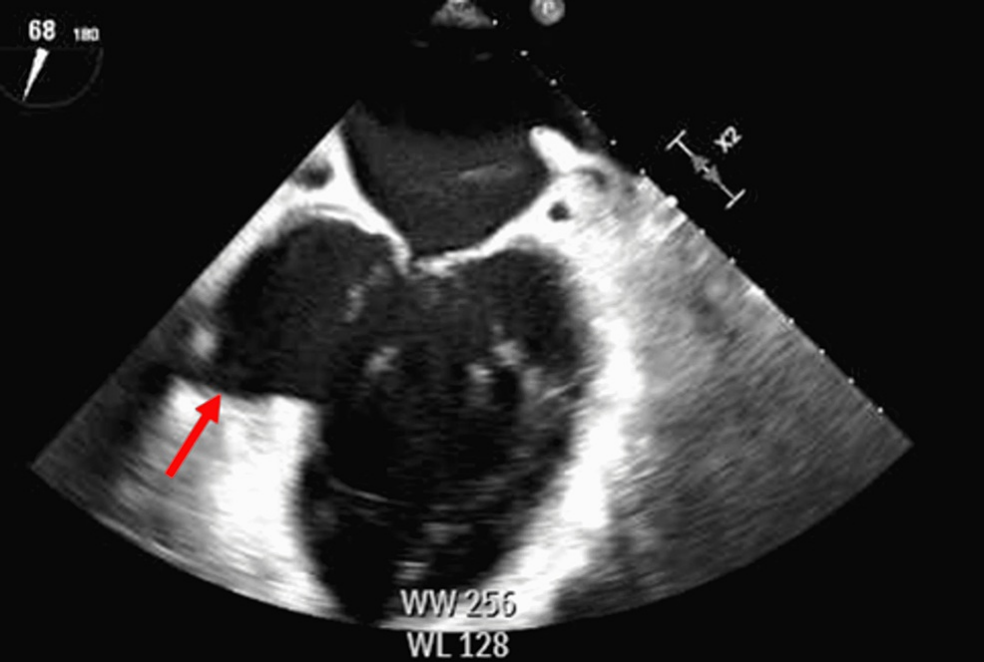
Left ventricle aneurysm with dilated left ventricle. Left ventricle aneurysm as seen by transesophageal echocardiogram (TEE) to help demonstrate the aneurysm, and the dilated left ventricle.

Further imaging with transthoracic echocardiogram (TTE) and transesophageal echocardiogram (TEE) revealed a dilated left ventricle with aneurysmal basal inferior, basal inferolateral, and mid inferolateral walls, seen in Figure 2 by TEE, as well as dilation of the right atrium. His left ventricular ejection fraction was estimated to be 45% compared to two years ago of 50%. The previous TTE showed no signs of the left ventricular aneurysm. The ejection fraction was possibly skewed due to flow into the aneurysm. Cardiac catheterization revealed the etiology of his heart failure due to total occlusion of the RCA, causing a posterior wall infarct aneurysm that was possibly worsened by the presence of a pre-existing VSD with a substantial left to right shunt seen in Video 1 which shows the VSD via bubble study. Video 2 further correlated the flow of the right to left shunt by color doppler. The patient was referred to surgery which he denied. He was then sent home on hospice care.

**Video 1:** Ventricular septal defect (VSD) shown by bubble study. VSD showing right to left shunt by bubble study.

**Video 2:** Ventricular septal defect (VSD) with color doppler. VSD showing right to left shunt by color doppler.

## Discussion

A VSD is a rare occurrence in the reperfusion era as well as a left ventricle aneurysm. These defects are generally considered to be mechanical complications post-myocardial infarction [6]. Even though these rarely occur anymore since the introduction of reperfusion therapy, when they do the mortality rate is high [4-6]. VSDs generally appear 3-5 days after myocardial infarction with symptoms of dyspnea and orthopnea. Left ventricle aneurysms appear much later post-myocardial infarction with a range of weeks to years with symptoms of heart failure.

Both VSDs and left ventricle aneurysms can present with an occlusion of the right coronary artery. It is rare in nature to see a left ventricle aneurysm or a VSD from occlusion of the right coronary artery, and even more rare to see the two together. Generally left ventricle aneurysms arise from occlusion of the left anterior descending artery, while a VSD can arise from multiple different infarcts [6].

The flow of blood observed in the TEE indicates a posterior septal bidirectional shunt through the VSD. While unclear if the right coronary artery was the main cause of the formation of the aneurysm, the bidirectional flow of blood through the VSD may have contributed to its formation. Blood flowing directly through the VSD against the posterior basal wall could have possibly caused mechanical changes to the anatomy of the heart as the posterior basal was weakened by the right coronary artery occlusion. Imaging of our patient showed direct communication between the left ventricle and the aneurysm having a wide neck.

The case presented differs from the typical presentation of VSDs in many respects including clinical presentation. Although the patient presented with signs of acute heart failure with severe lower extremity edema and shortness of breath, he did not present with a history consistent with an acute coronary event. Additionally, the patient had no indication in his outpatient medical records that he had experienced a myocardial infarction in the past. Another difference occurs in the diagnostic studies, in which the culprit vessel in the MI was the right coronary artery, rather than the left anterior descending artery.

Although our patient’s aneurysm is in the right coronary territory as might be expected, the size of the aneurysm is so large that one might have expected a clinical event to have been associated with its formation.

## Conclusions

We aimed to highlight the possibility of VSD and/or ventricular aneurysms without a history of MI and understand its manifestation relating to the mechanical changes from the flow of a VSD. The case presented showed the classic signs of post myocardial infarction mechanical changes in not one but two changes. It is an oddity as the possibility of the VSD causing turbulent flow could have caused or worsened the left ventricle aneurysm the patient presented with.
